# Extensive Bilateral Pulmonary Embolism Without Deep Vein Thrombosis After Varicose Vein Stripping: Superficial Venous Thrombosis as a Potential Source

**DOI:** 10.7759/cureus.107080

**Published:** 2026-04-15

**Authors:** Vismay Patel, Rubba S Khan, Ariel Litinski, Ajantha Rangasamy

**Affiliations:** 1 Internal Medicine, Hudson Regional Health (HRH) Bayonne University Hospital, Bayonne, USA; 2 Academy for Biotechnology, Morris County School of Technology, Denville, USA

**Keywords:** deep vein thrombosis (dvt), great saphenous vein, heparin drip, submassive pulmonary embolism, superficial vein thrombosis

## Abstract

Pulmonary embolism (PE) is a common and potentially life-threatening condition, most often arising from deep vein thrombosis (DVT). However, PE may rarely occur without demonstrable DVT, including after superficial venous procedures. We present the case of a 49-year-old morbidly obese woman with a recent history of right lower-extremity varicose vein stripping who presented with worsening shortness of breath. Imaging revealed a large burden of bilateral pulmonary emboli involving all lobar arteries. Duplex USG showed no evidence of DVT but demonstrated significant superficial venous thrombosis (SVT) in the great saphenous vein, with associated thrombus and hematoma. The patient remained hemodynamically stable and was managed with anticoagulation therapy. This case highlights SVT as a potential source of PE, particularly in the presence of additional risk factors such as obesity, recent surgery, and decreased mobility.

## Introduction

Pulmonary embolism (PE) is a major cause of morbidity and mortality worldwide and represents a significant component of venous thromboembolism (VTE), which includes both deep vein thrombosis (DVT) and PE. The annual incidence of VTE is estimated to be approximately 1 to 2 cases per 1,000 individuals, with PE accounting for a substantial proportion of related deaths [1]. In the United States, PE contributes to up to 100,000 deaths annually and remains an important cause of preventable in-hospital mortality [2].

In the majority of cases, pulmonary emboli originate from thrombi in the deep veins of the lower extremities, particularly the femoral and popliteal veins [3]. As such, diagnostic evaluation typically focuses on identifying DVT as the primary source. However, superficial venous thrombosis (SVT), historically considered a benign condition, has been increasingly recognized as a potential contributor to clinically significant thromboembolic events. Studies have demonstrated that approximately 20% to 25% of patients with SVT may have concurrent DVT or PE at the time of diagnosis [4].

A substantial proportion of patients with DVT have concomitant PE, with estimates ranging from 33% to 73%, while 51% to 97% of patients with PE have associated DVT.

The risk of embolization is particularly elevated when SVT involves the great saphenous vein, given its proximity to the deep venous system via the saphenofemoral junction [5]. Additionally, surgical interventions such as varicose vein stripping may predispose patients to thrombosis through endothelial injury, inflammation, and venous stasis. Although such procedures are generally considered low risk, thromboembolic complications may occur, particularly in patients with additional risk factors such as obesity, reduced mobility, and smoking [6].

We report a case of extensive bilateral PE in the absence of DVT following varicose vein stripping, highlighting SVT as a possible source of embolism.

## Case presentation

A 49-year-old morbidly obese female, height 5'8" and weight 133 kg, with a BMI of 44.6, presented to the ED with a one-week history of progressively worsening shortness of breath, which acutely intensified on the day of admission. She reported difficulty breathing while preparing for work and was unable to continue her usual activities. On initial presentation, vital signs were BP 129/61 mmHg, heart rate 61 bpm, and oxygen saturation 97% on room air. However, shortly thereafter, the patient developed tachycardia with a heart rate of 105 bpm and required supplemental oxygen at 6 L via nasal cannula to maintain oxygen saturation of 94%.

Three weeks prior to presentation, she had undergone varicose vein stripping of the right lower extremity. Since the procedure, she reported decreased mobility, although she continued to work intermittently in a predominantly sedentary role. Approximately five days before presentation, she noted localized swelling and possible infection at the surgical site, which was treated with antibiotics.

She denied chest pain, fever, chills, recent travel, use of oral contraceptive pills, or any prior history of thromboembolic events. She also denied any personal or family history of hypercoagulable disorders. Her past surgical history included gastric sleeve surgery, hysterectomy, and tonsillectomy. She was not taking any regular medications and reported chronic tobacco use and daily alcohol consumption.

On presentation, the patient was tachycardic with a heart rate of 105 beats per minute and required supplemental oxygen at 6 L via nasal cannula to maintain an oxygen saturation of 94%. Physical examination revealed decreased breath sounds bilaterally and tenderness along the right lower extremity.

**Figure 1:**
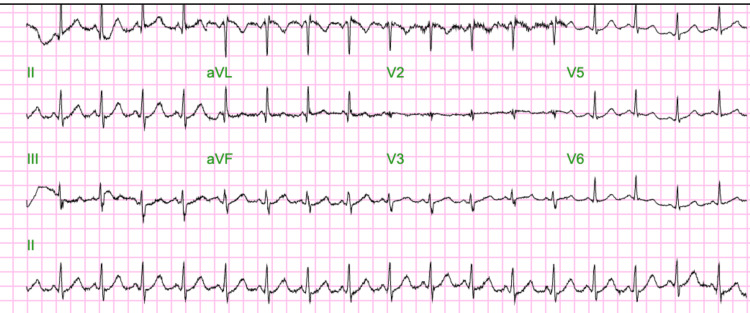
Electrocardiogram demonstrating sinus tachycardia without evidence of the classic S1Q3T3 pattern. No significant ST-segment elevation or depression is observed. The findings are consistent with nonspecific changes.

Electrocardiography demonstrated sinus tachycardia without evidence of an S1Q3T3 pattern or right heart strain (Figure 1).

**Figure 2:**
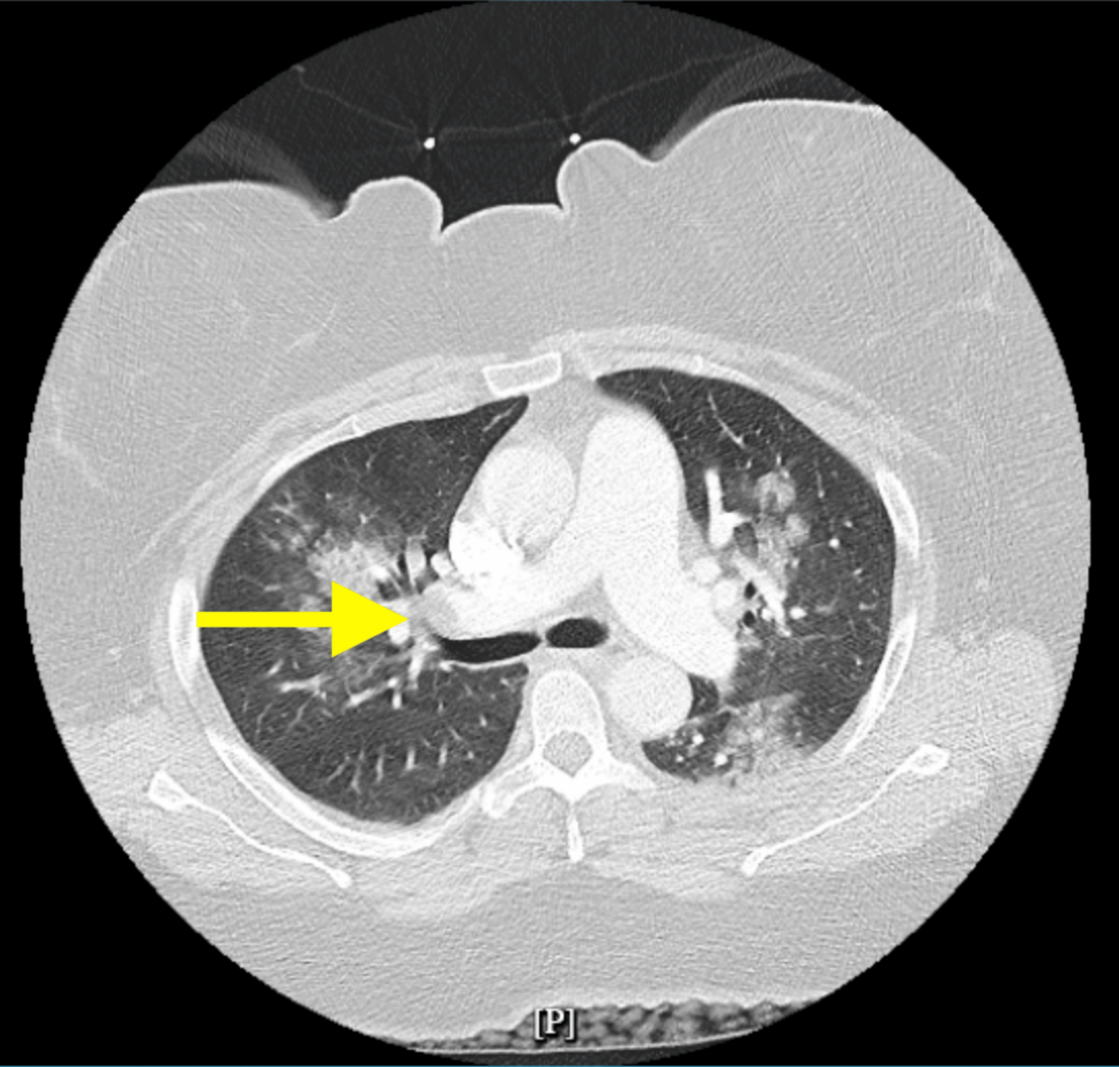
Computed tomography angiography (CTA) of the chest revealed extensive bilateral pulmonary emboli involving all lobar arteries, with intraluminal filling defects within contrast-opacified pulmonary vessels. Additional findings included ground-glass opacities suggestive of pulmonary infarction or inflammatory change, as well as enlargement of the main pulmonary artery consistent with pulmonary hypertension.

Computed tomography angiography (CTA) of the chest revealed extensive bilateral pulmonary emboli involving all lobar arteries, with intraluminal filling defects noted within contrast-opacified pulmonary vessels. Additional findings included ground-glass opacities suggestive of pulmonary infarction or inflammatory changes, as well as enlargement of the main pulmonary artery consistent with pulmonary hypertension (Figure 2).

**Figure 3:**
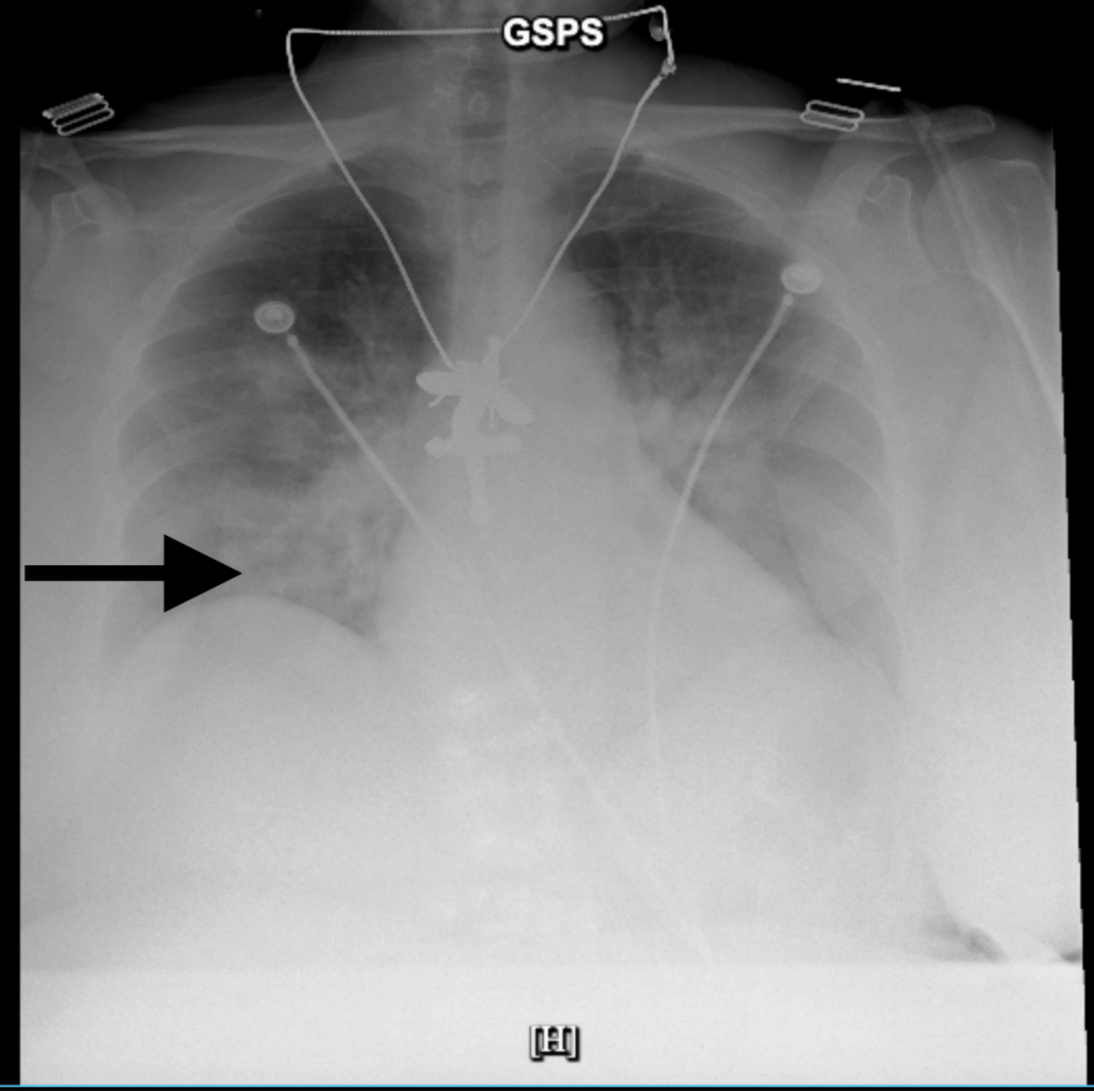
Chest radiograph demonstrating bilateral perihilar airspace opacities and consolidation.

Chest radiography demonstrated bilateral perihilar airspace opacities, with differential considerations including pulmonary edema, multifocal pneumonia, or early acute respiratory distress syndrome. The bilateral infiltrates were likely secondary to pulmonary infarction related to embolism and did not significantly alter management. They improved over the next 3 days (Figure 3).

**Figure 4:**
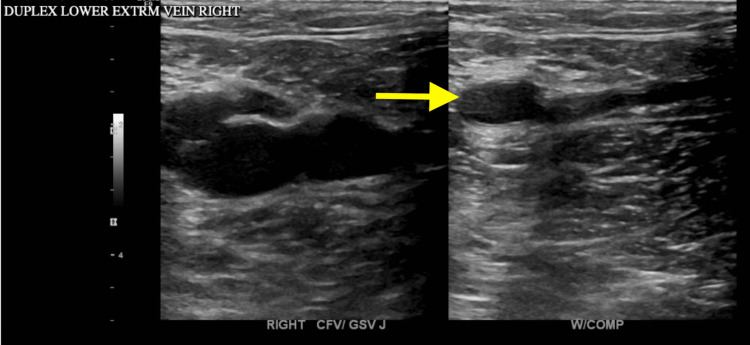
Duplex USG of the right lower extremity demonstrated superficial venous thrombosis of the great saphenous vein with a non-compressible, thrombosed lumen. The deep venous system remained compressible, with no evidence of deep vein thrombosis.

Duplex USG of the right lower extremity demonstrated SVT of the great saphenous vein, while the deep venous system of the lower extremity was compressible without evidence of DVT. Evaluation was limited to lower-extremity venous ultrasound, and the iliac veins and inferior vena cava were not directly assessed (Figure 4).

Laboratory evaluation revealed an elevated D-dimer level, mild leukocytosis, and elevated cardiac biomarkers, including a B-type natriuretic peptide (BNP) of 605 pg/mL and troponin levels decreasing from 0.08 ng/mL to 0.03 ng/mL, consistent with right ventricular strain in the setting of PE.

Key laboratory findings are summarized in Table 1.

**Table 1:** Laboratory results demonstrated elevated D-dimer levels consistent with thrombosis, stable coagulation parameters except for anticoagulation-related changes in activated partial thromboplastin time, mild leukocytosis, and elevated cardiac biomarkers suggestive of right heart strain. Lactate levels were normal, and glucose was mildly elevated.

Test	Result	Reference Range
D-dimer	4380 ng/mL	<500 ng/mL
Prothrombin time (PT)	13.0 sec	11-13.5 sec
International normalized ratio (INR)	1.17	0.8-1.2
Activated partial thromboplastin time (aPTT)	197.3 → 95.1 sec	25-35 sec
WBC count	9 → 17.0 × 10⁹/L	4-11 × 10⁹/L
Troponin	0.08 → 0.03 ng/mL	<0.04 ng/mL
B-type natriuretic peptide (BNP)	605 pg/mL	<100 pg/mL
Lactate	0.9 mmol/L	0.5-2.0 mmol/L
Glucose	110 mg/dL	70-99 mg/dL

Venous blood gas analysis demonstrated a pH of 7.42, pCO₂ of 51 mmHg, and HCO₃ of 29.8 mmol/L, consistent with compensated respiratory acidosis. While acute PE more commonly results in respiratory alkalosis due to hyperventilation, this patient’s findings may reflect hypoventilation, possibly related to obesity, increased work of breathing, or evolving respiratory fatigue. The elevated bicarbonate suggests a compensatory metabolic response, indicating a more chronic or subacute process rather than an acute acid-base disturbance.

**Figure 5:**
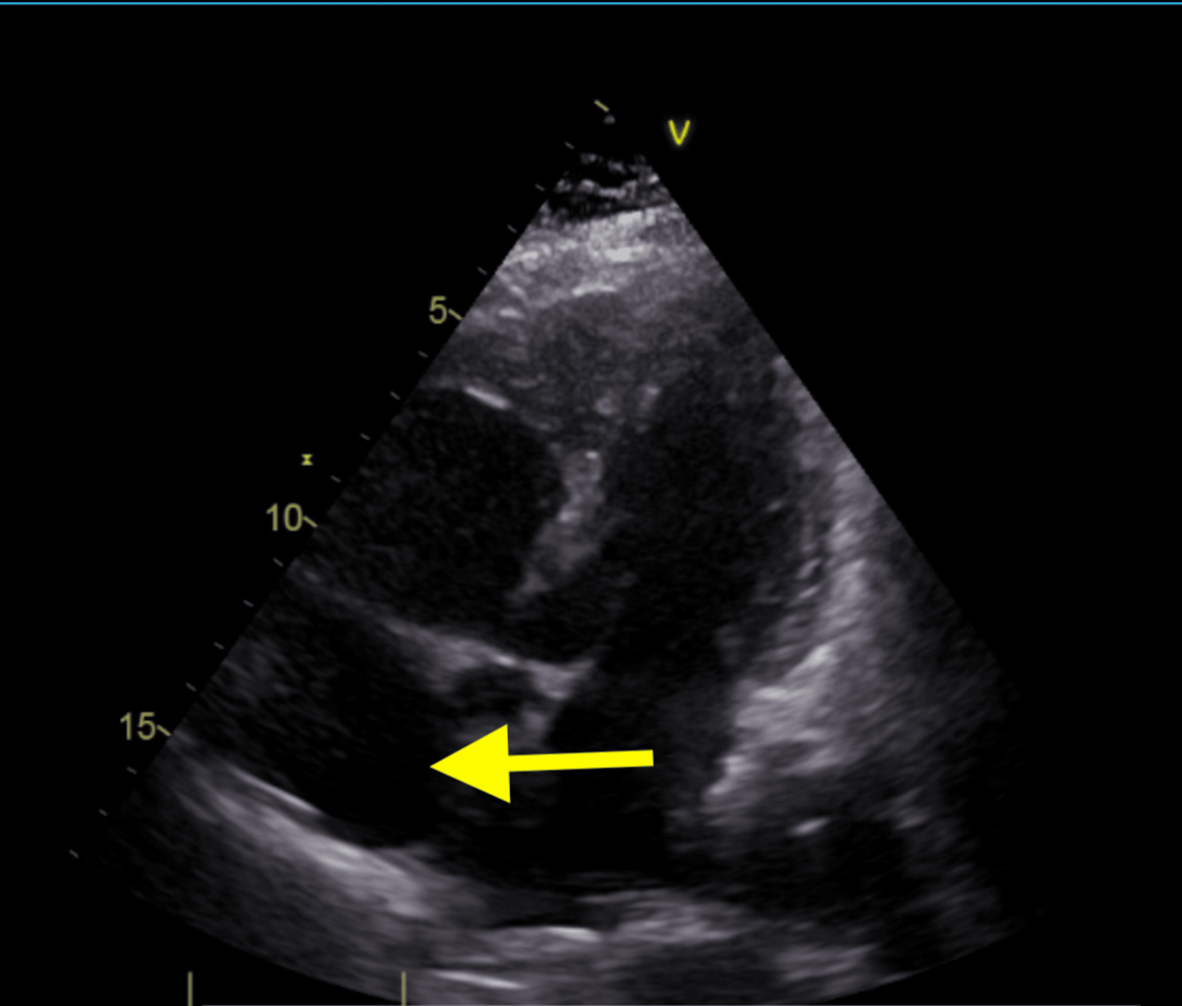
Transthoracic echocardiography demonstrated mild right ventricular dilation with reduced systolic function, moderate right atrial enlargement, severe pulmonary hypertension, and severe tricuspid regurgitation, while left ventricular size and systolic function were preserved, with normal segmental wall motion.

Transthoracic echocardiography demonstrated mild right ventricular dilation with reduced systolic function, moderate right atrial enlargement, and severe tricuspid regurgitation. The estimated pulmonary artery pressure was 74 mmHg, consistent with severe pulmonary hypertension. Left ventricular size and systolic function were preserved, with an ejection fraction of 72% (Figure 5).

The patient was diagnosed with submassive PE and was started on IV heparin therapy for two days. Given her hemodynamic stability, she was not a candidate for thrombolysis or thrombectomy and was subsequently transitioned to apixaban 10 mg BID on day 2. Her symptoms improved during hospitalization, and she was discharged with outpatient follow-up for hematologic evaluation and ongoing anticoagulation management.

## Discussion

This case is unique in demonstrating extensive bilateral PE in the absence of detectable DVT following varicose vein stripping, reinforcing SVT as a clinically significant embolic source. While PE is classically associated with thrombi originating from the deep venous system, this case supports the growing evidence that SVT may serve as a source of emboli.

Differential diagnoses considered in this case included acute decompensated heart failure, pneumonia, acute respiratory distress syndrome, and chronic thromboembolic pulmonary hypertension. The absence of significant left ventricular dysfunction, lack of infectious symptoms, and imaging findings consistent with intraluminal filling defects on CTA supported PE as the primary diagnosis.

In this patient, no DVT was identified on duplex imaging, yet extensive pulmonary emboli were present. The detection of thrombus within the great saphenous vein provides a plausible source of embolism; however, a definitive causal relationship cannot be established. This finding aligns with prior studies demonstrating a significant association between SVT and VTE [4,5].

Although this event may initially appear unprovoked, the patient had multiple contributing risk factors, including recent venous surgery, reduced mobility, obesity, and tobacco use. These factors collectively fulfill Virchow’s triad and likely contributed to thrombus formation and subsequent embolization. It remains possible that an undetected DVT had already embolized prior to imaging, and therefore SVT should be considered a likely but not definitive source.

Despite the large clot burden, the patient remained hemodynamically stable, consistent with intermediate-risk PE. Current guidelines at the time of writing the report recommend anticoagulation as the primary treatment in such cases, with escalation of therapy reserved for patients with clinical deterioration [7,8].

The presence of right ventricular dysfunction and pulmonary hypertension reflects the physiological impact of the embolic burden and warrants close follow-up. Right ventricular dysfunction has been reported in up to 30% to 50% of patients with PE and is associated with worse outcomes [9]. This case further emphasizes that SVT should not be considered a benign condition, particularly in high-risk patients or in the postoperative setting [10,11].

## Conclusions

PE can occur in the absence of DVT and may originate from superficial venous thrombosis, which should be considered a potential source, particularly in the appropriate clinical context. This case highlights the importance of recognizing SVT as a potentially clinically significant condition rather than a benign entity. Patients with additional risk factors, including obesity and reduced mobility, may be at increased risk of thromboembolic complications. Early diagnosis, appropriate anticoagulation, and close clinical follow-up are essential to prevent adverse outcomes. Increased awareness of this association may help guide timely evaluation and management in similar clinical scenarios.
